# Biosorption of silver cations onto *Lactococcus lactis* and *Lactobacillus casei* isolated from dairy products

**DOI:** 10.1371/journal.pone.0174521

**Published:** 2017-03-31

**Authors:** Maciej Milanowski, Paweł Pomastowski, Viorica Railean-Plugaru, Katarzyna Rafińska, Tomasz Ligor, Bogusław Buszewski

**Affiliations:** 1 Department of Environmental Chemistry and Bioanalytics, Faculty of Chemistry, Nicolaus Copernicus University, Toruń, Poland; 2 Interdisciplinary Centre of Modern Technologies, Nicolaus Copernicus University, Toruń, Poland; Tallinn University of Technology, ESTONIA

## Abstract

The current work deals with the phenomenon of silver cations uptake by two kinds of bacteria isolated from dairy products. The mechanism of sorption of silver cations by *Lactococcus lactis* and *Lactobacillus casei* bacteria was investigated. Inductively coupled plasma–mass spectrometry (ICP-MS) was used for determination of silver concentration sorbed by bacteria. Analysis of charge distribution was conducted by diffraction light scattering method. Changes in the ultrastructure of *Lactococcus lactis* and *Lactobacillus casei* cells after treatment with silver cations were investigated using transmission electron microscopy observation. Molecular spectroscopy methods, namely Fourier transform-infrared spectroscopy (FT-IR) and matrix-assisted laser desorption/ionization mass spectrometry (MALDI MS) were employed for description of the sorption mechanism. Moreover, an analysis of volatile organic compounds (VOCs) extracted from bacterial cells was performed.

## Introduction

Lactic acid bacteria (LAB) are gram-positive, facultatively anaerobic, usually nonmotile and nonsporulating bacteria, whose characteristic feature is production of lactic acid from carbohydrates by means of fermentation as their major end product [1, 2]. This capability and other features have led to the wide use of LAB in numerous industrial applications. The most important ones are: production of fermented foods using starter cultures and utilization as probiotics [3–6]. Moreover, LAB have been recognized in the field of bioremediation, especially when bacterial strains act as sorbents [7–9]. *Lactobacillus acidophilus* has been employed for arsenic(III) removal from waste water [10]. Cadmium and lead ions can be bound by certain species of *Lactobacillus* and *Bifidobacterium* [11]. Chromium resistant *Lactobacillus* strains have displayed their ability to reduce chromium(VI) to chromium(III) [12]. Furthermore, *Lactobacillus casei* was found to be the most effective binding agent of Cu^2+^ amongst several tested microorganisms [13]. Also, biosorption mechanism of silver cations by *Lactobacillus* sp. strain A09 was investigated in the study of Lin et al. [14].

Nowadays, there are broad industrial and medical applications of silver nanoparticles (AgNPs), including electronics, food industry, clothing, medical devices and cosmetics [15]. This indicates continuously increasing and widespread usage of silver nanoparticles. AgNPs are in electrochemical equilibrium with silver cations [16], hence their high demand causes increased amount of this element being released into the environment, especially into the aquatic one. Therefore, the problem of its removal is a top issue [17–21] and LAB are microorganisms which can be used for uptake of silver cations [22]. In this field it is crucial to understand the mechanisms of biosorption of Ag by bacteria species capable of binding silver cations.

The aim of this study was to develop a methodology for biosorption of silver cations by/onto isolates of lactic acid bacteria. For this purpose, (i) isolation of selected LAB obtained from milk products (e.g. milk, cheese) was conducted; (ii) identification of isolated bacteria by sequencing 16S rRNA genes, as well as intact cell MALDI-TOF MS was performed; (iii) microbiological profile description of LAB was carried out; (iv) biosorption of silver ions on selected LAB was employed and (v) determination of silver binding mechanisms was realized with the use of multiple techniques.

## Materials and methods

### Instrumentation

NanoDrop 2000c (Thermo Fisher Scientific, Wilmington, DE, USA) was used to measure concentration of DNA. For polymerase chain reaction (PCR) amplification we employed Mastercycler^®^ pro thermocycler (Eppendorf, Hamburg, Germany) and electrophoresis was carried out with the use of PowerPac^™^ Universal Power Supply (Bio-RAD Laboratories, Hercules, CA, USA). Identification of the isolated bacteria and investigation of silver biosorption were both performed with the use of ultrafleXtreme MALDI-TOF/TOF mass spectrometer (Bruker Daltonik, Bremen, Germany). Concentration of silver was determined with the use of CX 7500 ICP-MS spectrometer (Agilent Technologies, Santa Clara, CA, USA). FT-IR SPECTRUM 2000 used for acquisition of IR spectra was purchased from PerkinElmer (Waltham, MA, USA). The zeta potential was analyzed by Zetasizer Nano Series analyzer (Malvern Instruments, Malvern, United Kingdom). For transmission electron microscopy we used Tecnai F20 X-Twin system (FEI Europe, Eindhoven, The Netherlands). The employed ultramicrotome was Leica EM UC7 (Leica Microsystems, Wetzlar, Germany). We also used Axio Observer D1 fluorescence microscope (Carl Zeiss, Oberkochen, Germany). GC/MS analyses were carried out using an Agilent 6890A gas chromatograph (Agilent Technologies, Santa Clara, CA, USA) coupled with an Agilent 5975 Inert XL MSD mass spectrometer (Agilent Technologies). The system was equipped with a CP-Porabond-Q 25 m × 0.25 mm × 3 μm column (Agilent Technologies). Extractions of volatile organic compounds (VOCs) were performed using 75 μm Carboxen/polydimethylsiloxane (PDMS) fiber (Supelco, Bellefonte, PA, USA).

### Materials

Various combinations of media were employed for bacteria cultivation, namely: Tryptic Soy Broth (TSB; Soybean-Casein Digest Medium; Bacto, Sydney, Australia), Tryptic Soy Agar (TSA; Soybean-Casein Digest Agar Medium; Bacto), de Man, Rogosa and Sharpe Agar (MRSA; Sigma Aldrich, Steinheim, Germany), de Man, Rogosa and Sharpe (MRS) Bulion and M17 Bulion (Merck, Darmstadt, Germany). EXTRACTME DNA BACTERIA kit (version: 1.2014; DNA-Gdańsk, BLIRT, Gdańsk, Poland) was used to isolate LAB. Taq PCR Master Mix Kit (Qiagen, Hilden, Germany) together with Ideal Marker DNA M10Kpz (DNA-Gdańsk, BLIRT, Gdańsk, Poland) were applied for PCR reaction. Electrophoresis was conducted using RNase-Free Water (Qiagen), agarose (MAXIMUS, Łódź, Poland), 1× TAE buffer (Bioline, London, United Kingdom) and ethidium bromide (AppliChem, Darmstadt, Germany). QIAquick PCR Purification Kit (Qiagen) was applied for PCR products purification. For transmission electron microscopy we used 1× phosphate-buffered saline (PBS) buffer, glutaraldehyde, ethanol, uranyl acetate and lead citrate purchased from Sigma Aldrich. MTP Anchor Chip 384 target (Bruker Daltonik, Bremen, Germany) was used in MALDI-TOF MS experiments, as well as chemicals from Sigma Aldrich: LC-MS grade Chromasolv water, ethanol, acetonitrile, trifluoroacetic acid (TFA), isopropanol, and products from Avantor Performance Materials (Gliwice, Poland): acetone, hydrochloric acid and methanol. Matrices for MALDI analyses were α-cyano-4-hydroxycinnamic acid (HCCA) and 2,5-dihydroxybenzoic acid (DHB) (both from Sigma Aldrich). KCl and NaCl buffers (both from Avantor Performance Materials) were purchased for zeta potential measurements. Other chemicals and consumables such as: Ag solution, KBr, acridine orange and CaF_2_ plate were acquired from Sigma Aldrich. 22 mL headspace crimp top vials and polytetrafluoroethylene (PTFE)/butyl septa for HS-SPME-GC/MS experiments were purchased from PerkinElmer (Waltham, MA, USA). Ultra-pure water from a Milli-Q water system (Millipore, Bedford, MS, USA) was used throughout the work.

Milk products (milk, cheese, cottage cheese, whey, powdered milk, powdered whey) were supplied by Dairy Factory in Drzycim and Piotrków Kujawski, Poland.

### Isolation of bacteria from milk products

Samples of milk, cheese, cottage cheese, whey and powdered milk were plated on three culture media, namely TSA, MRSA and M17, and incubated at 37°C for 24 h. Then, the same combination of media was streaked with the obtained biological material using a sterile inoculation loop and incubated at 37°C for 24 h. Subsequently, the grown colonies were applied for preparation of dilutions in the range of 10^−1^ to 10^−8^ using sterilized 0.87% KCl and double distilled water (H_2_O_dd_). All dilutions were plated (1 mL of inoculum) onto Petri dishes with culture media, MRSA and M17, respectively. The plates were placed into an incubator for 24 h at 37°C.

### Microbiological characteristics of LAB

Enumeration of the total number of microorganisms was performed according to the method described in the Polish norm PN-A-86034-04:1993. A series of sequential dilutions (1 mL of material: 9 mL of H_2_O/KCl) was prepared from the initial dilution (10 mL of milk: 90 mL of H_2_O/KCl). The first dilution was made by transferring 1 mL of a sample to 9 mL of H_2_O/KCl which made it 10^−2^ dilution of the original sample. Immediately after the 10^−2^ dilution was shaken, the vessel was uncapped and 1 mL was aseptically transferred to a second 9 mL-portion of H_2_O/KCl solution. As a consequence, the second blank represented a 10^−3^ dilution of the original sample. Shaking the 10^−3^ dilution vigorously and transferring 1 mL of it to the third 9 mL-portion of water/KCl resulted in obtaining 10^−4^ dilution blank. The process was continuously repeated until the dilution of 10^−7^ was reached. All samples (1 μL of suspension) were plated in duplicate onto Petri dishes filled with M17 and MRSA media and then incubated at 37°C.

### Identification of the isolated bacteria by 16S PCR

Isolation of DNA from 27 selected bacteria was conducted using the EXTRACTME DNA BACTERIA kit. Samples were prepared according to the manufacturer’s protocol. Concentration of DNA was measured using NanoDrop 2000c within the range of 10.8 to 22.8 ng μL^-1^, and, subsequently, 1 μL of each DNA sample was transferred into a vial. 2 μL of Taq PCR Master Mix Kit was added to each sample together with 1 μL of F1 primers (5′-GAG TTT GAT CCT GGC TCA G-3′) and R12 primers (5′-ACG GCT ACC TTG TTA CGA CTT-3′). Finally, the samples were placed in Mastercycler^®^ pro thermocycler with the following program: initial denaturation at 95°C for 2 min followed by 30 cycles of denaturation at 94°C for 1 min, annealing at 55°C for 1 min and extension at 72°C for 2 min with the final extension at 72°C for 5 min. The PCR amplification fragments were resolved by agarose (1.0%) gel electrophoresis at 100 V for 30 min. The gel was stained with ethidium bromide, and the bands were visualized under UV light. Then, the PCR products were purified and sequenced according to Hrynkiewicz et al. [23]. At first, the PCR products were purified using the QIAquick PCR Purification Kit. Then, the PCR primers acted as sequencing primers in the process of direct sequencing of PCR products. The sequences were aligned manually aided by the Sequencher system (TW Version 5.1, Gene Codes, Ann Arbor, MI, USA). Comparison of the obtained sequences of all isolates was performed using the BLAST database of the National Center for Biotechnology Information (NCBI).

### Identification of the isolated bacteria by intact cell MALDI-TOF MS

The matrices: HCCA (10 mg mL^-1^) and DHB (50 mg mL^-1^) were prepared in Bacterial Solution (EtOH/ACN/H_2_O, 1:1:1 (v/v/v)). Trifluoroacetic acid (TFA) solution was added to the Bacterial Solution to reach the final concentration of 2.5% v/v. Then, under sterile conditions, two loopfuls (approximately 10 mg) of bacterial cells were suspended in 5 μL of Bacterial Solution and thoroughly vortexed for 30 s. 2 μL of bacterial suspension was mixed with 2 μL of a matrix and then 1 μL of the mixture was spotted on a ground steel MALDI target. After 30 min, when all spots had dried, the target was placed in the ultrafleXtreme MALDI-TOF/TOF mass spectrometer for a measurement according to Pomastowski et al. [24]. The ultrafleXtreme mass spectrometer was equipped with a modified Nd:YAG laser (smartbeam II^™^) operating at the wavelength of 355 nm and the frequency of 2 kHz. Intact cell (IC) MALDI TOF MS spectra were recorded manually in linear positive mode within *m*/*z* range of 300–30000 and applying the acceleration voltage of 25 kV. All mass spectra were acquired and processed with the dedicated software: flexControl and flexAnalysis, respectively (both from Bruker).

### Biosorption of silver onto *L*. *lactis* and *L*. *casei*

10 mL of M17 and MRSA was inoculated with *L*. *lactis* and *L*. *casei*, respectively. Three solutions of silver cations at concentrations of 1 mg L^-1^ (1 ppm), 0.1 mg L^-1^ (0.1 ppm) and 0.01 mg L^-1^ (0.01 ppm) were made from a stock solution of silver (60 mg L^-1^ AgNO_3_),. After addition of silver cations, each of the inoculated solutions was measured for optical density (OD) with incubation lasting for 24 h at 37°C. The following MALDI-TOF MS experiments were conducted in order to determine changes in spectra between bacteria alone (control) and bacteria treated with silver cations (1 ppm, 0.1 ppm and 0.01 ppm). The settings and conditions of the measurements using the ultrafleXtreme mass spectrometer were identical as described above.

Independently, 5 mL (at 10^6^ cells/mL) of inoculum was prepared. Then, 1 mL of inoculum was transferred to three flasks with TSB medium. One of them served as a control. 2.5 mL of a stock solution of Ag^+^ was added to the second flask (OPTION 1) at the time of inoculation. All flasks were then incubated in a water bath (37°C) with shaking (150 rpm) for 24 h. After this period 2.5 mL of Ag^+^ stock solution was transferred into the third flask (OPTION 2) and all flasks were subsequently incubated under the same conditions for 48 h. Finally, all the samples were centrifuged for 15 min (14500 rpm). The separated supernatants and the pellets were stored in a refrigerator (4°C) and used for further analyses.

### Inductively Coupled Plasma—Mass Spectrometry (ICP-MS)

The obtained supernatants from OPTION 1 & 2 were transferred into sterile Eppendorf tubes (500 μL). The pellets from OPTION 1 & 2 were suspended in 5 mL of sterile H_2_O and centrifuged again for 15 min (14500 rpm) to purify the biomass from the medium. Next, the supernatant was removed and the pellet was suspended in 500 μL of H_2_O. 300 μL of HNO_3_ and 100 μL of HCl were added to the suspension and incubated in a thermomixer (90°C) with shaking for 4 h until the precipitate was completely dissolved. Each sample was examined for the presence of silver using inductively coupled plasma—mass spectrometry (ICP-MS) with a quadrupole mass analyzer.

### Fourier Transform Infrared spectroscopy (FT-IR)

The bacterial pellets from OPTION 1 & 2 were suspended in 250 μL of H_2_O and thoroughly centrifuged. One drop of the suspension (120 μL) was transferred onto acetone-cleaned plate of CaF_2_ and dried at 105°C for approximately 2 min. IR spectra were acquired in the range of 4000–400 cm^-1^ and 20 scans were carried out for each sample. The measurements were performed using FT-IR SPECTRUM 2000.

### Zeta (ζ) potential measurement

The measurement of zeta potential was carried out with different solvents: 0.7% KCl (pH 2–11) and 0.7% NaCl (pH 3–9). The bacterial pellet from OPTION 2 was suspended in 1 mL of H_2_O and vortexed thoroughly until obtaining a homogeneous suspension of bacteria. Then, 980 μL of a buffer with a specific pH was added to 20 μL of bacteria solution and mixed by pipetting. The whole sample was vortexed for 30 s and incubated for 1 min in an ultrasonic bath. Thus prepared samples were placed directly in a cuvette and all measurements were recorded using the Zetasizer Nano Series analyzer. The ζ-potential values were provided directly by the instrument. The measurements were carried out at 25°C in triplicate for 10 cycles. The obtained results were shown in diagrams using the sigmoidal model available in the CurveExpert Professional 2.0 software (Hyams Development, USA).

### Transmission Electron Microscopy (TEM)

Selected samples from the control and OPTION 2 were measured using Tecnai F20 X-Twin electron microscope coupled with Energy Dispersive X-ray (EDX) detector. First, a portion of 10 mL of culture broth was centrifuged, washed twice with sterilized phosphate-buffered saline, and the biomass was subjected to transmission electron microscopy. Bacterial cells were fixed with 2% glutaraldehyde for 4 h. After eliminating the remaining glutaraldehyde, dehydration process was conducted with 20, 30, 40, 50, 60, 70, 80, 90, and 100% ethyl alcohol. Fixed cells were then infiltrated and embedded in LR Gold resin. The embedded material was cut on Leica EM UC7 ultramicrotome into ultra-thin sections (50 nm) and placed on grids coated with formvar. Ultrathin sections were subsequently stained with 2.5% uranyl acetate and 0.4% lead citrate solutions, and examined using Tecnai F20 X-Twin system.

### Fluorescence microscopy analysis

Fluorescence microscopy examination was performed via acridine orange/ethidium bromide (AO/EB) staining in order to discriminate living and dead cells. Membrane integrity visualization was detected through the filter set at 43 He (excitation 550/25, emission 605/70) and 38 (excitation 470/40, emission 520/50) using Axio Observer D1 fluorescence microscope. Bacteria cells of *L*. *casei* and *L*. *lactis* grown in TSB medium were treated with AgNO_3_ (1 ppm). Two methods (OPTION 1 & 2) were applied for both strains. In the first method (OPTION 1) AgNO_3_ (1 ppm) was added to cell culture medium (1×10^6^ of cells) and analysis was performed after 1 h and 24 h of incubation. In the second method (OPTION 2) the inoculum of bacteria (1×10^6^ of cells) was first incubated at 37°C for 24 h, then treated with AgNO_3_ (1 ppm) and analyzed after 1 h and 24 h of incubation at 37°C. In both cases cells were stained with AO/EB with the final concentration of 0.12/0.4 μg mL^-1^ and incubated at room temperature for 5 min in the dark. Cells were then centrifuged and the remaining supernatant was discarded to eliminate the unbound dyes. Next, the cell pellet was resuspended in a small volume of PBS-1X. Control samples were prepared basing on cells untreated with AgNO_3_ (1 ppm). All experiments were performed in a dark room to avoid photobleaching of the dyes.

### Gas Chromatography—Mass Spectrometry (GC/MS)

Empty glass vials were stored at 60°C for 2–3 days, and then removed from a dryer and left to stand for about 15 minutes to equilibrate with the surroundings. 2 g of the bacterial pellet from OPTION 2 was weighed, transferred into a vial and capped. The headspace air was pumped out twice through a syringe to generate underpressure. VOCs were extracted and analyzed using headspace solid phase microextraction gas chromatography—mass spectrometry (HS-SPME-GC/MS). Volatile compounds were collected using 75 μm Carboxen/PDMS fiber at 50°C for 15 min. Gas chromatography was performed with helium as the carrier gas with the flow rate of 1.4 mL min^-1^; the temperature of the split-splitless injector was set at 200°C. The oven temperature program was as follows: the initial 40°C were kept for 2 min, and ramped at 10°C min^-1^ to 140°C, then again ramped at 5°C min^-1^ to 270°C and kept for 5 min. Acquisition was performed at the mass range of 30–300 *m*/*z*. Spectra were collected for ions generated by electron ionization (EI) at 70 eV; both the ion source and the transfer line temperature was set at 200°C.

## Results

### Isolation and characteristics of bacteria isolated from milk products

Bacteria were isolated from the investigated milk products and were described by visible morphological features. Isolate colonies appearing in plates were classified according to their shape, color, texture, size, etc. S1 Table shows selected 27 bacteria strains along with a description of the applied medium, diluent, and a brief characterization. M17 and MRSA proved to be the best media for the culture of lactic acid bacteria owing to the greatest number of grown colonies on the plates. On the other hand, the type of a diluent (H_2_O or KCl) used for preparation of inoculum had no significant effect on the growth of the bacteria.

### Identification of the obtained LAB by 16S PCR and intact cell MALDI-TOF MS

Sequences obtained from 16S rRNA gene sequence analysis were used to search for similarities using the BLAST (basic local alignment search tool; http://blast.ncbi.nlm.nih.gov/Blast.cgi) program. Identity of the representative isolates were determined on the basis of the highest scores (typically ≥ 95%). The results of this study showed that analysis with 16S rRNA gene sequencing could provide successful identification for the investigated LAB species, *L*. *lactis* (100%) and *L*. *casei* (100%) (Table 1).

**Table 1:** Results of 16S rRNA sequencing of *L*. *lactis* and *L*. *casei* isolated from milk products.

Name	Identified strain	BLAST	Overlap (%)
Cheese isolated from MRSA HOH yellow dark, medium: M17	*Lactobacillus casei*	*Lactobacillus casei* [KF673500]	1430/1430 (100%)
		*Lactobacillus paracasei* subsp. *paracasei* [KF418817]	1430/1430 (100%)
		*Lactobacillus casei* [JN560891]	1430/1430 (100%)
Cow milk from Świecie district, Poland	*Lactococcus lactis*	*Lactococcus lactis* [JN7852396]	1433/1433 (100%)

**Fig 1:**
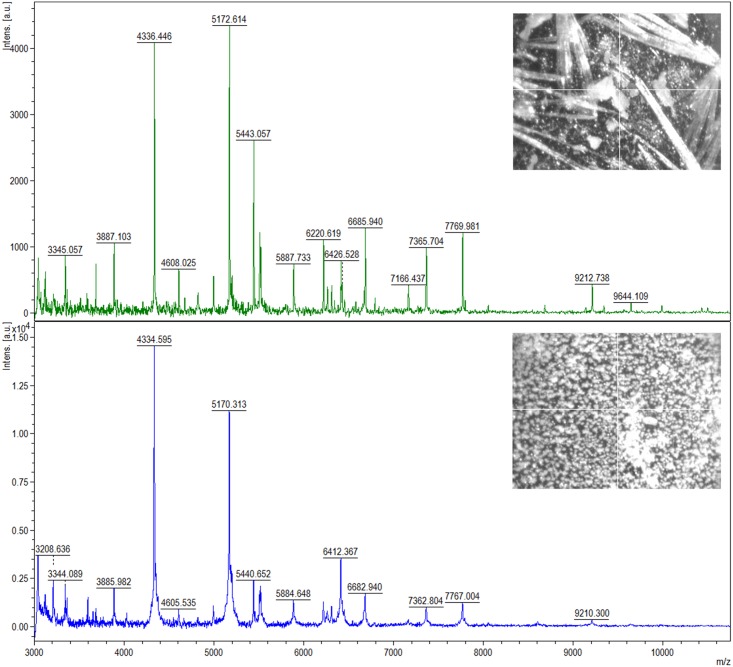
A spectrum of *L*. *lactis* recorded using MALDI-TOF MS technique with the use of DHB (top) and HCCA (bottom) matrix; photos of crystals of the matrices are shown in the corner.

Two bacterial strains: *L*. *lactis* and *L*. *casei*, were chosen for identification using the ultrafleXtreme MALDI-TOF/TOF mass spectrometer. The acquired spectra were compared with MALDI spectra of reference bacterial strains: *L*. *lactis* ATCC 49032 and *L*. *casei* ATCC 334 and the coverage of 99%– 100% was obtained. Moreover, in order to select appropriate conditions for the analysis, we tested and compared two different matrices: DHB and HCCA. As portrayed in Fig 1 a greater number of signals (i.e. 70) was recorded for *L*. *lactis* when α-cyano-4-hydroxycinnamic acid (HCCA) was used as a matrix, in comparison to application of 2,5-dihydroxybenzoic acid (DHB) where only 43 ions were detected.

**Fig 2:**
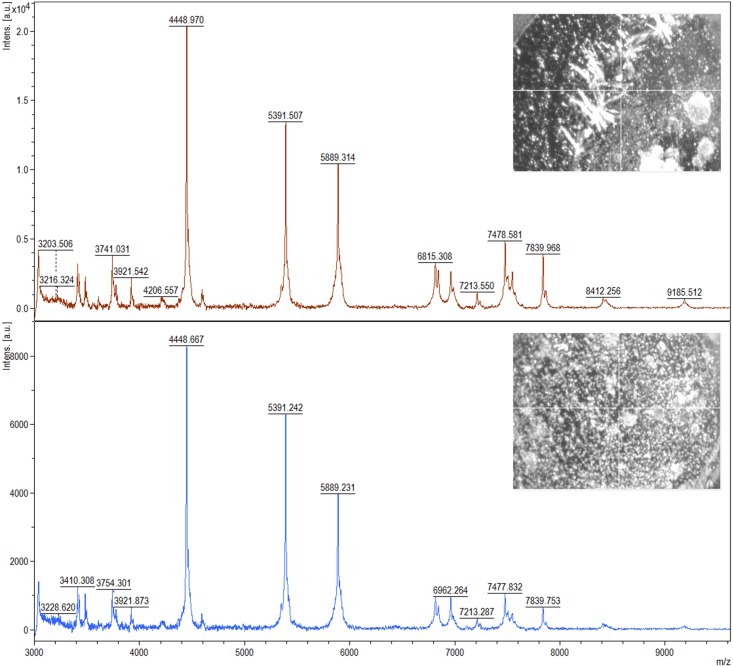
A spectrum of *L*. *casei* recorded using MALDI-TOF MS technique on DHB (top) and HCCA (bottom) matrix; photos of crystals of the matrices are shown in the corner.

In the case of *L*. *casei*, we recorded 78 signals for DHB matrix. HCCA gave only a slightly smaller number of signals (74) on the obtained spectra (Fig 2).

Based on the value of the signal to noise ratio and intensity of individual peaks, it can be concluded that HCCA matrix is a better choice in the case of bacteria *L*. *lactis*. On the contrary, better quality of spectra was obtained using DHB matrix for *L*. *casei*. The optimal starting optical density of microbial cells was in the range of 0.3–0.4. Among the dilutions– 1:1, 1:10 and 1:100 the best mass spectra were recorded for 1:1 dilution. MS spectra obtained for the studied species at this dilution factor were characterized by the highest intensity, high signal to noise ratio and the largest number of registered peaks. Therefore, in order to prepare reference spectra we used the 1:1 dilution of bacterial cells in HCCA matrix.

### Biosorption of silver onto selected LAB evaluated by MALDI-TOF MS

**Fig 3:**
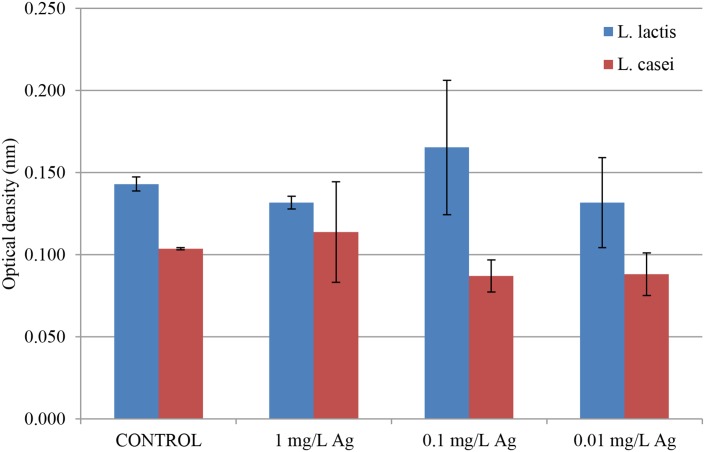
OD values for the studied strains of bacteria inoculated with different concentrations of silver.

Fig 3 shows OD values obtained after 24 h of incubation of *L*. *lactis* and *L*. *casei* with 1 mg L^-1^ (1 ppm), 0.1 mg L^-1^ (0.1 ppm) and 0.01 mg L^-1^ (0.01 ppm) of silver cations.

Native strains served as a control. There were no significant differences in the amount of bacterial cells expressed by their optical density in the case of both *L*. *lactis* and *L*. *casei*. None of the applied concentrations of Ag^+^ affected the final OD, moreover the number of cells remained relatively constant in comparison with the control. Hence, in this study the concentration of silver cations was specially selected so as not to be cytotoxic to the isolated lactic acid bacteria and not to inhibit their growth.

**Fig 4:**
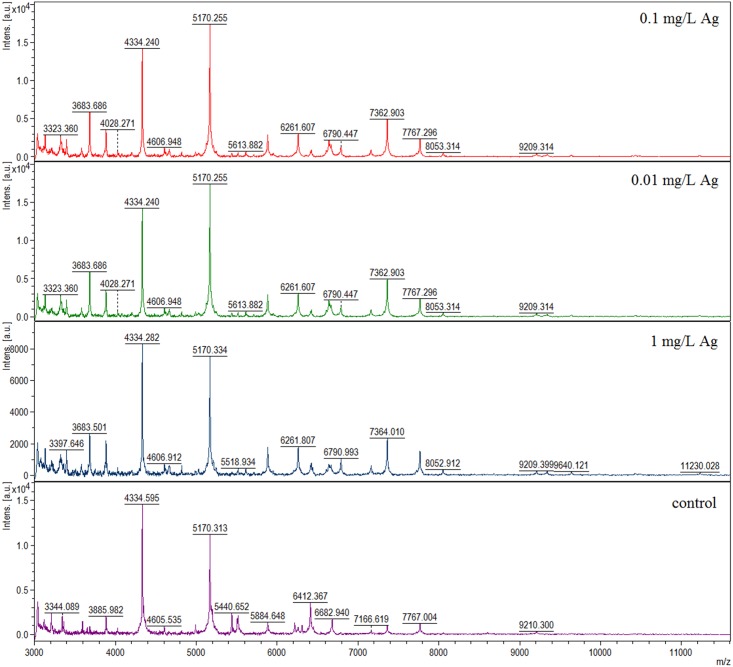
MALDI-TOF MS spectra of *L*. *lactis* with different concentrations of silver compared to a reference spectrum of these bacteria.

In order to determine changes in the cells of LAB after silver binding MALDI-TOF MS spectra from bacteria inoculated with silver were compared with previously obtained reference spectra of *L*. *lactis* and *L*. *casei*. Fig 4 illustrates that MALDI-TOF MS spectra derived from silver-enhanced *L*. *lactis* were significantly different than those originating from a native strain.

Seven signals appeared to have significantly increased intensity in samples containing silver. This effect was observed for the peaks at 3683.607, 3885.089, 5170.334, 5884.648, 7166.619, 7362.804 and 7767.004 *m/z*. Moreover, intensity of the peaks at 5440.652 and 5518.837 *m/z* were increased at the control spectrum. Fourteen signals were recorded only for samples with silver cations. They were: 3133.449, 3212.085, 3322.476, 3397.050, 3583.491, 4820.593, 6261.807, 6424.261, 6640.434, 6790.993, 8053.314, 9338.727, 9640.121 and 11230.028 *m/z*. On the other hand, eight signals were present exclusively for *L*. *lactis* without any modifications: 3119.572, 3208.636, 3344.089, 3594.997, 6217.919, 6308.829, 6412.367 and 6682.940 *m/z*. These peaks were absent from MS spectra of the samples with the addition of silver. For *L*. *casei* no changes in MALDI-TOF MS spectra were observed.

### ICP-MS measurement

**Fig 5:**
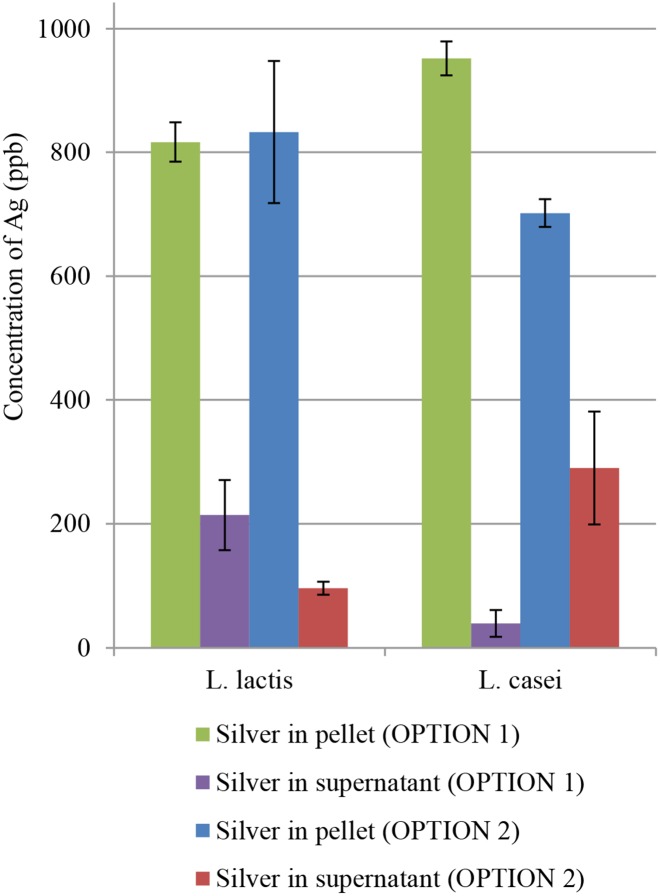
Concentration of Ag in OPTION 1 & 2 measured using ICP-MS.

Fig 5 illustrates differences in the content of silver for particular variants (OPTION 1 & 2) in the tested bacterial cells before and after inoculation with silver.

In the case of *L*. *lactis*, the content of silver in both variants (OPTION 1 & 2) remained at a similar level of about 79% which means that *L*. *lactis* bacteria bound similar amount of silver regardless of the time of Ag^+^ addition. This observation confirms the thesis that Ag^+^ in the concentration of 1 ppm has no significant effect on the physiology of *L*. *lactis* and the ability of this strain to adopt Ag^+^. In the case of *L*. *casei*, in turn, significant differences in the processes of Ag^+^ binding were observed. Bacteria supplemented with silver cations 24 h after inoculation (OPTION 2) bound 70.77% ± 3.21% of the element as compared to bacteria incubated with silver at the time of inoculation (OPTION 1) where sorption was at the level of 95.96% ± 2.89%. The results indicate an ability for adaptation of *L*. *casei* grown in an environment abundant in silver cations. Moreover, the performed study demonstrated higher adsorption efficiency of bacterial cells inoculated at the time of addition of silver cations.

### FT-IR spectroscopy

Spectral characteristics of native and modified LAB was conducted to find active chemical groups involved in the silver-binding process. S1 and S2 Figs show FT-IR spectra of native strains and strains after modification resulting from addition of silver. In the case of *L*. *lactis* a broad peak localized in the region of 1530–1630 cm^-1^ was observed upon the addition of silver to the sample (S1 Fig), which is characteristic for samples with Ag addition. The absorption band at 1456 cm^-1^ was flattened in OPTION 2. Moreover, signals at 1059 and 1236 cm^-1^ became deeper in the case of silver-treated *L*. *lactis*. For *L*. *casei*, in turn, the only observed change was a broad peak in the region of 1530–1630 cm^-1^ in OPTION 1 (S2 Fig).

### Determination of zeta (ζ) potential

**Fig 6:**
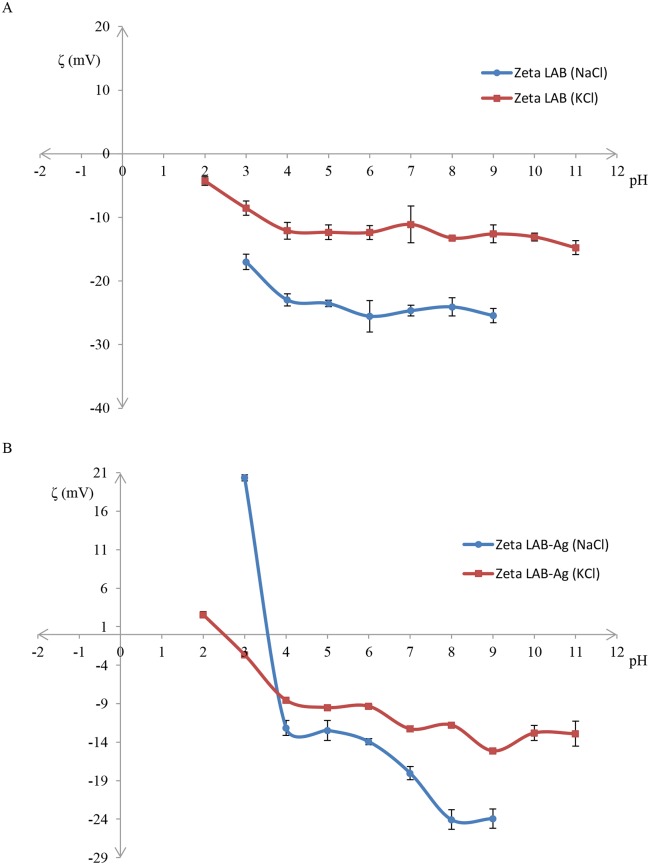
Zeta potential of bacterial cells of *L*. *lactis* in different solutions, and obtained before (A) and after (B) silver addition.

S3(A) Fig for *L*. *lactis* shows the size of a bacterial cell within 1000–5000 nm in each of the used buffers (KCl and NaCl) and at all tested pH values. The graph (B) displays the same peak with reduced intensity and an additional weak signal from a particle of about 5000–10000 nm in size. The following charts show changes in the value of the zeta potential resulting from changes in pH of a buffer (Fig 6).

Fig 6(A) illustrates charge distribution of native *L*. *lactis*. The zeta potential of *L*. *lactis* suspended in 0.7% KCl in pH range of 2–11 oscillated between –4 and –15 mV. In the case of 0.7% NaCl, a decrease of zeta potential was observed (ranging from –17 to –25 mV) in pH range of 2–11. Fig 6(B) shows zeta potential values for *L*. *lactis* after sorption of Ag^+^. Depending on the buffer applied, the zeta potential for *L*. *lactis* with sorbed Ag^+^ ranged from +20 to –24 mV for NaCl buffer, and from +3 to –16 mV for KCl buffer. There was a sharp decrease in the potential values registered for both buffers: from +20 mV to –12 mV in the case of NaCl buffer and from +3 mV to –3 mV for KCl buffer. Values of the isoelectric point of *L*. *lactis* with sorbed Ag^+^ were approximately 3.5 and 2.5 for NaCl and KCl buffers, respectively. After the drop, constant values of ζ potential for KCl and its continuous decrease until –24 mV for NaCl buffer were observed.

### TEM imaging

**Fig 7:**
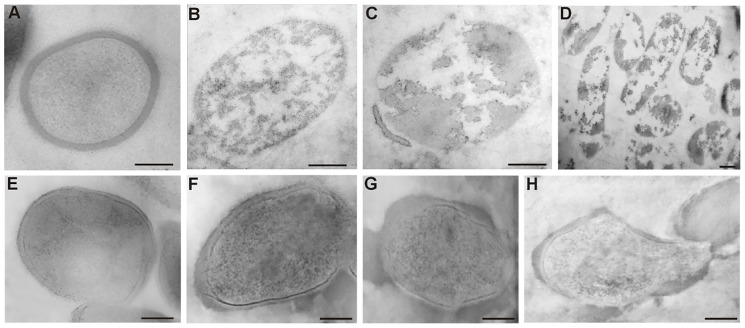
Ultrastructure of bacterial cells of *L*. *lactis* and *L*. *casei*. (A)–native *L*. *lactis*, (B), (C), (D)–*L*. *lactis* cells after 48 hours of incubation with silver cations (OPTION 2), (E)–native *L*. *casei*, (F), (G), (H)–*L*. *casei* cells after 48 hours of incubation with silver cations (OPTION 2). Bar: 200 nm.

In order to investigate changes in the ultrastructure of *L*. *lactis* and *L*. *casei* cells after treatment with Ag^+^, a transmission electron microscopy observation was performed (Fig 7).

Control cells of *L*. *lactis* not treated with AgNO_3_ (Fig 7(A)) have an oval shape, homogenous cytoplasm and a relatively thick cell wall. After 48 hours of incubation with Ag^+^, the ultrastructure underwent characteristic changes (Fig 7(B), 7(C) and 7(D)). Electron light bands were visible on the area of the cytoplasm. The cell wall was present only in the form of individual pieces on single cells, whereas most of the observed cells were completely deprived of it. There was no continuity of the cell membrane, either.

Cells of *L*. *casei* are oval in shape and have a homogenous cytoplasm and a smooth surface (Fig 7(E)). After incubation with AgNO_3_ their ultrastructure did not undergo such significant changes as in the case of *L*. *lactis*. Thickening and uneven surface of cell walls was a characteristic change (Fig 7(F), 7(G) and 7(H)). Their cytoplasm was still electron dense, although much less homogeneous. Only in a few cells of *L*. *casei* changes in the shape and separation of cell membrane from the cell wall were observed. These results suggest that AgNO_3_ has a limited effect on the ultrastructure of *L*. *casei* cells.

### Fluorescence microscopy examinations with AgNO_3_

**Fig 8:**
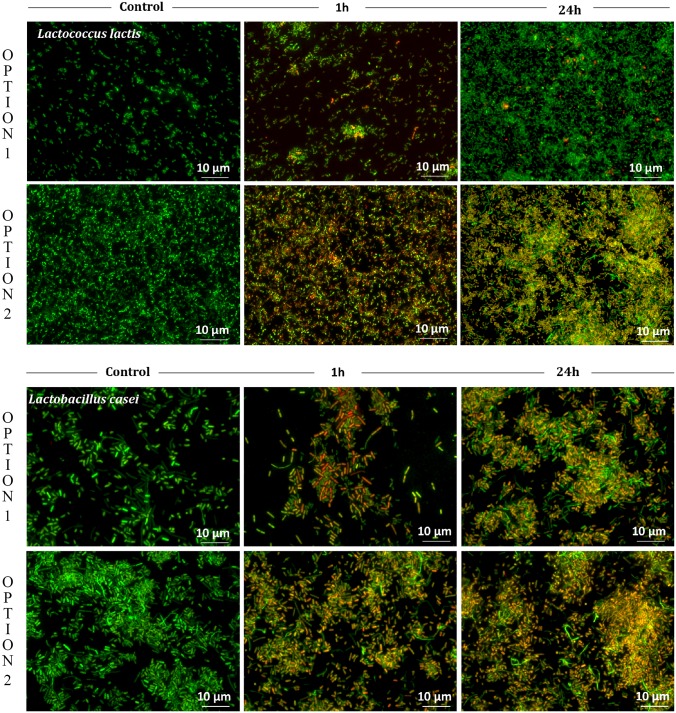
Comparative viability of *L*. *lactis* and *L*. *casei* cells after the treatment with AgNO_3_ (1 ppm) regarding the first (OPTION 1) and the second method (OPTION 2).

For visualization of *L*. *lactis* and *L*. *casei* viability after the treatment with AgNO_3_ (1 ppm) we performed staining with nucleic acid dyes (acridine orange and ethidium bromide). The living cells that showed only green fluorescence and the dead ones which displayed red color are shown in Fig 8.

In the case of the first method (OPTION 1), only viable cells displaying green fluorescence were visible in the samples of *L*. *lactis* and *L*. *casei* at the time of inoculation. After 1 h of incubation with AgNO_3_, dead cells for both bacterial species could be observed. However, specific differences were noticeable after 24 h of incubation. In the case of *L*. *casei*, the number of dead cells displaying red fluorescence was much higher than the number of those displaying green fluorescence which suggests that *L*. *casei* is not as resistant to the used concentration of silver cations as *L*. *lactis*. An ability for adaptation of cells to Ag cations was recorded for *L*. *lactis*, which was manifested by a high number of cells displaying green fluorescence, which in turn indicated that under these conditions the majority of cells were alive. For the second method (OPTION 2) (Ag^+^ added 24 h after inoculation) the results were comparable regardless of the chosen bacteria. After 1 h and 24 h of incubation with silver cations a growing number of dead cells could be noticed. The number of dead cells surpassed the number of live cells, hence the toxic effect of AgNO_3_ (1 ppm) was clearly visible.

### HS-SPME-GC/MS experiment

**Fig 9:**
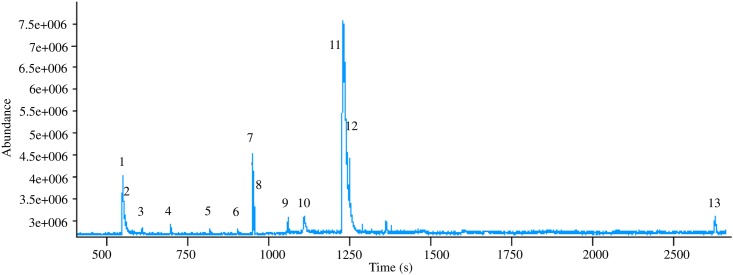
Chromatogram of VOCs from *L*. *lactis* obtained using HS-SPME-GC/MS, where: (1) acetaldehyde, (2) ethanol, (3) butane, (4) acetone, (5) 2-methylpropanal, (6) ethenyl acetate, (7) 2-butanone, (8) ethyl acetate, (9) hexane, (10) 3-methylbutanal, (11) 1-pentanol, (12) 3-methyl-1-butanol, (13) 2-phenylethyl acetate.

HS-SPME-GC/MS analysis of bacterial headspace resulted in identification of 13 volatiles. The chromatogram acquired for *L*. *lactis* is illustrated in Fig 9.

Among compounds found in the headspace of *L*. *lactis* the most abundant were: 1-pentanol, 2-phenylethyl acetate and 2-butanone. Other compounds, such as: ethyl acetate, hexane, acetaldehyde and 2-methylpropanal could also be distinguished.

## Discussion

### Isolation and identification of bacteria from milk products

Due to their widespread distribution, lactic acid bacteria can be isolated from different biological matrices, including raw milk, dairy products and fermented foods. Other sources of lactobacteria are fecal samples, soil and plants [25, 26]. In particular, samples of human and animal milk are habitats rich in nutrients and they are often used for isolation of a diverse spectrum of LAB [27–29]. In this field, growth media such as MRSA and M17 are often employed for cultivation of LAB [30, 31]. In our study, identification of *L*. *lactis* and *L*. *casei* was carried out by two different techniques, namely: 16S rRNA gene sequencing and intact cell MALDI-TOF MS. The first technique is commonly used for identification of LAB [32, 33]. However, a growing interest of researchers in methods utilizing MALDI-TOF MS has been recently observed, due to high sample throughput, repeatability and precision of this technique. Currently, MALDI-TOF MS profiling of whole bacterial cells is used more frequently for bacterial identification [34, 35]. In order to select optimal conditions for analysis we tested and compared two matrices: HCCA and DHB. The former one proved to be more appropriate for *L*. *lactis*, whereas the latter one—for *L*. *casei*. This could result from different nature of the matrices. HCCA forms fine crystals, whereas DHB is characterized by large needles (Figs 1 and 2). This feature causes specific alterations in the extraction mechanism of bacterial components [24].

### Biosorption of silver

In general, biosorption is a process of removal of substances from a solution by biological material [36]. It is a widely used technique for elimination of heavy metals (e.g. Cd, Pb, Cu, Cr) from waters by live or dead biomass and cellular products [37, 38]. The processes of biosorption involve, inter alia, absorption, adsorption, ion exchange, surface complexation and precipitation [36]. There are various types of biosorbents including bacteria, fungi, yeasts, algae, as well as industrial and agricultural waste [37, 39]. For example, bacteria of *Lactobacillus* sp. strain A09 show silver binding properties [14]. Recently, brewery fermentation industry waste yeast has been employed to adsorb Ag(I) from an aqueous solution [40]. Our study revealed that also *L*. *lactis* and *L*. *casei* isolated from milk and its products are good biosorbents of silver cations as they absorbed about 70–96% of silver from 1 ppm solution. The rate of silver cations uptake was the highest for *L*. *casei* when Ag^+^ was added at the time of inoculation. There is a report that bacteria originating from metal-containing waters or sediments show greater tolerance to heavy metals than those from uncontaminated environments [41]. The obtained results indicate that this strain of bacteria very quickly adapts to the presence of silver cations in the environment when the culture is at the stationary growth phase. However, when silver cations were added to bacteria at the logarithmic phase of growth the rate of uptake was significantly reduced. It suggests that cells in this phase of growth are very susceptible to the toxic effect of silver.

Furthermore, we observed several changes in MALDI-TOF MS spectra recorded for silver-treated *L*. *lactis* in comparison to a reference spectrum of a native strain. We registered increased intensity of some ions and additional signals for samples containing silver. These alterations are an obvious result of the presence of a stressing agent i.e. silver cations. They are a well-known bactericide and their activity against multidrug-resistant, highly pathogenic or probiotic bacteria has been extensively described [42, 43]. The statement regarding stressing influence of silver on bacteria is supported by a recent work of Chudobova et al. who investigated the effect of addition of metal ions (including Ag^+^) on acquired MALDI-TOF MS spectra of bacterial proteome [44]. They studied modifications in *Staphylococcus aureus* protein composition and registered additional peaks in the obtained spectra suggesting metabolic changes of heavy metal resistant *S*. *aureus*. These signals were connected with changes in protein morphology due to the metal influence. Such alterations in the metabolic profile of bacteria were also observed in the study of Gopal et al. This group proposed a mechanism for interaction of five nanoparticles (of Ag, NiO, Pt, TiO_2_ and ZnO) with *S*. *aureus* and *Pseudomonas aeruginosa*. Significant changes in the number and intensity of signals on MALDI-MS spectra of both bacterial strains were reported after a treatment with AgNPs, and in comparison with a control sample of native bacteria [45]. Antibacterial properties and influence of graphene-based silver nanoparticles (AgNPs—GE) on *P*. *aeruginosa* were investigated in the work of He et al. These researchers performed MALDI—TOF/TOF MS analysis to find changes in proteomic profile induced by AgNPs—GE and AgNO_3_. Untreated bacteria served as a control. They found the total of 28 proteins which were altered in expression following the exposure of *P*. *aeruginosa* to AgNPs—GE and AgNO_3_ [46]. This is another confirmation of the thesis that addition of silver influences metabolic and proteomic profiles of bacteria, which can be monitored using MALDI-TOF MS technique.

### Further analyses of LAB-Ag

FT-IR spectroscopy is as an advanced tool for characterization of molecular composition which allows characterization of conformationally distinct structures in biological molecules [47]. S1 Fig shows a broad absorption peak formed after addition of silver, which is assigned to amide II and amide I vibrations of β-pleated sheet structures of proteins [48, 49]. The disappearing peak at 1456 cm^-1^ in OPTION 2 may indicate C-H deformation of >CH_2_ group in lipid proteins [47–49]. Two bands at 1059 and 1236 cm^-1^ in silver-treated *L*. *lactis* resulted from the presence of C-O-C and C-O groups dominated by ring vibrations in various polysaccharides and components of proteins contributing to amide III band, respectively [47, 50]. Hence, it is possible that carboxyl groups of proteins are involved in the process of biosorption of silver. As can be seen in S2 Fig, addition of Ag to *L*. *casei* caused formation of a broad peak in the region of 1530–1630 cm^-1^, which could be attributed to amide II vibrations [48, 49].

S3(A) Fig demonstrates that regardless of the used buffers and the applied pH values the size of bacterial cell of *L*. *lactis* was registered in the range of 1000–5000 nm. Broadening of the base of the peak and a new signal observed in the range of 5000–10000 nm (S3(B) Fig) indicate formation of aggregates of bacterial cells resulting from the presence of electrostatic interactions on the surface or attachment of an additional molecule [51, 52]. This is directly connected with the obtained results from the zeta potential measurements illustrated in Fig 6(B) for LAB-Ag. Bacteria suspended in NaCl had a more negative potential value as compared to bacteria suspended in KCl. Hence, bacteria suspended in NaCl showed greater dispersion stability. This phenomenon is caused by repulsive forces between solvated biocolloids of bacterial cells generated by sodium ions [51, 53]. Potassium ions have a greater ionic radius than sodium ions, which reduces the shielding effect between biocolloids [54]. When analyzing influence of pH of the system a sharp decrease of zeta potential was noticed for pH 2–4 which was a result of progressive deprotonation of carboxyl groups of bacterial structural components. For pH 5–8 a constant value of zeta potential could be observed which reflected the proteolytic balance [53, 55]. Bacteria after biosorption of Ag^+^ show higher electric charge. In the case of NaCl, bacteria at pH 2–3 acquired charge of about +20 mV; for KCl this charge was +3 mV. The increased value of ζ potential indicated reduced dispersion stability and destabilization of the biocolloidal system. LAB-Ag demonstrated reduced stability of biocolloids as compared to native cells. This was a consequence and also an evidence of the biosorption processes of silver cations on the surface of bacterial cells [22, 53, 55, 56].

Ultrastructural TEM analysis of bacterial strains *L*. *lactis* and *L*. *casei* treated with AgNO_3_ is portrayed in Fig 7. The changes ((B), (C), (D)) prove that the applied silver concentration had destructive influence on the ultrastructure of *L*. *lactis* cells and led to their death. In the case of *L*. *casei* ((F), (G), (H)), the observed increase in wall thickness can suggest a mechanism preventing penetration of silver cations into the cell and the site of immobilization. These processes are probably utilized to protect the fragile protoplast from the toxic effects of silver, and thus they can increase the ability of cell sorption. Thickening of the cell walls is a phenomenon that has already been observed in gram-positive bacterium *S*. *aureus* in response to the presence of vancomycin. It is suggested that this process prevents penetration of antibiotic molecules into cells [57]. The changes in ultrastructure of LAB cells suggest the uptake of silver cations and no reduction of silver cations at the concentration of 1 ppm, which is illustrated in Fig 3.

Fig 8 demonstrates viability of *L*. *lactis* and *L*. *casei* after treatment with AgNO_3_ (1 ppm). At the stationary phase of growth (OPTION 1) *L*. *lactis* appeared to be more resistant to the applied concentration of silver than *L*. *casei*, which suggest adaptation to this specific environment. Silver cations added to *L*. *lactis* and *L*. *casei* cells, which were at the logarithmic growth phase, induced a toxic effect—the number of dead cells was significantly higher than the one of live cells. It can be found in the literature that *L*. *lactis* was tested in terms of its ability to adapt to different conditions, such as heat shock, osmotic stress, low pH, starvation [58], and other stressing factors [59–62]. These alterations often changed the physiology of bacteria, hence it is possible that the presence of silver influences molecular and metabolic processes of the tested *L*. *lactis*, as portrayed in Fig 8.

Fig 9 shows a profile of volatile organic compounds for *L*. *lactis* obtained using HS-SPME-GC/MS. This technique is widely used for extraction of VOCs from different matrices, including headspace of bacterial strains. For example, it was employed to absorb the volatile compounds from headspace of *Helicobacter pylori* [63], *Clostridium difficile* [64], and many others [65, 66]. Volatile organic compounds were extracted by analyzing food-related products such as milk [67], cheese [68], kefir-like beverages [69] and bushera (Ugandan traditional fermented beverage) [70] inoculated with *L*. *lactis* and other bacterial strains. In the headspace of *L*. *lactis* we found volatile compounds originating from natural metabolism of bacteria. VOCs such as acetone, butane, ethanol, acetaldehyde and 3-methylbutanal were detected in numerous studies using HS-SPME-GC/MS technique [71–74]. Hence, our methodology seems to be appropriate for observation of changes of bacterial metabolism.

## Conclusions

Milk and its products are sources of LAB resistant to the presence of silver below 100 ppm and capable of its biotransformation and assimilation. We found that two investigated lactic acid bacteria, *L*. *lactis* and *L*. *casei*, had the ability to sorb silver cations which was most likely associated with the cell surface of bacterial strain evaluated using TEM examinations. In our study we demonstrated changes in spectra obtained from MALDI-TOF MS profiling of whole bacterial cells when using different concentrations of silver. ICP-MS analyses proved that *L*. *casei* could grow in the environment of silver cations and that these bacteria could bind more of this element if it was added at the time of inoculation. Such dependence was not observed in the case of *L*. *lactis*. The bacteria bound nearly the same amount of silver during inoculation and upon addition after 24 hours. FT-IR spectroscopy and ζ potential measurements showed that several groups, mostly carboxyl groups, were involved in binding and biotransformation of silver cations. Also profiling of volatile organic compounds performed for *L*. *lactis* demonstrated a variety of compounds in bacterial headspace which were typical products of bacterial metabolism. Finally, it can be stated that the employed combination of various instrumental methods provided new possibilities for interpretation of physicochemical phenomena occurring at the interface of bacteria—bacteria and bacteria—metal—bacteria.

## Supporting information

S1 Table Bacteria isolated from milk products. (DOCX)

S1 Fig FTIR spectra of native L. lactis and modified strain (OPTION 1 & 2) with silver. (TIF)

S2 Fig FTIR spectra of native L. casei and modified strain (OPTION 1 & 2) with silver. (TIF)

S3 Fig The size of bacterial cell from L. lactis in NaCl buffer and at pH = 3 obtained before (A) and after (B) silver addition. (TIF)
