# Supplementary material for: Biosorption of silver cations onto Lactococcus lactis and Lactobacillus casei isolated from dairy products
Source: PLoS One. 2017 Mar 31;12(3):e0174521. doi: 10.1371/journal.pone.0174521 (PMC5375156; doi:10.1371/journal.pone.0174521)
Supplement: S1 Table — (DOCX) [file pone.0174521.s001.docx]

**S1 Table. Bacteria isolated from milk products.**

| No | Name | Comments |
| --- | --- | --- |
| 1 | Milk II isolated from M17 HOH small pink, medium: MCK | white, convex, glistening, with a slight mucus surface |
| 2 | Milk I isolated from M17 HOH white transparent, medium: M17 | white, milky, glistening |
| 3 | Cheese isolated from MRSA HOH yellow dark, medium: M17 | small, white, transparent, glistening |
| 4 | Milk I isolated from M17 HOH small yellow, medium: M17 | small, white, transparent, glistening |
| 5 | Milk I isolated from M17 HOH yellow spot, medium: M17 | yellow with brighter edge, convex, glistening |
| 6 | Milk I isolated from MRSA KCl beige spot, medium: M17 | small, beige, convex, glistening |
| 7 | Cheese isolated from MRSA HOH white, medium: MRSA | small, white, transparent, glistening |
| 8 | Milk I isolated from M17 HOH white transparent, medium: MH2 | small, white, transparent, convex, glistening |
| 9 | Milk II isolated from M17 HOH big spot, medium: M17 | bright, yellow, milky, flat, glistening |
| 10 | Milk II isolated from M17 HOH big spot, medium: MRSA | bright, yellow, milky, flat, glistening |
| 11 | Milk I isolated from MRSA KCl, beige spot, medium: MRSA | small, beige, transparent, convex, glistening |
| 12 | Milk I isolated from M17 HOH, yellow spot, medium: MRSA | white, milky, convex, glistening |
| 13 | Cheese isolated from MRSA HOH, yellow, medium: MRSA | white, yellow, dull |
| 14 | Milk I isolated from M17 HOH, yellow spot, medium: MH2 | yellow with brighter edge, convex, glistening |
| 15 | Milk I isolated from M17 KCl small pink, medium: M17 | small, bright, yellow, glistening |
| 16 | Milk I isolated from M17 KCl small pink, medium: MH2 | small, white, yellow, glistening |
| 17 | Milk I isolated from MRSA KCl, beige spot, medium: MRSA | small, beige, pink, glistening |
| 18 | Milk II isolated from MRSA HOH small white, medium: MRSA | small, transparent, convex, glistening |
| 19 | Milk II isolated from M17 HOH small pink, medium: MH2 | yellow, pink, convex, glistening |
| 20 | Milk II isolated from M17 HOH big spot, medium: MH2 | milky, yellow, convex, glistening |
| 21 | Milk I isolated from MRSA KCl pink spot, medium: MH2 | small, beige, milky, convex, dull |
| 22 | Cheese isolated from MRSA HOH pink, medium: MRSA | small, beige, glistening |
| 23 | Cheese isolated from MRSA HOH yellow dark, medium: MRSA | small, beige, white, glistening |
| 24 | Cottage cheese isolated from MRSA HOH, medium: MRSA | beige, pink, milky, flat, glistening |
| 25 | Cheese isolated from MRSA HOH yellow dark, medium: MRSA | small, white, dull, convex |
| 26 | Cottage cheese isolated from MRSA HOH, medium: M17 | small, white, beige, convex, glistening |
| 27 | Cheese isolated from MRSA HOH big spot, medium: M17 | beige, yellow, milky, dull |
