# Supplementary figures and images for: Biosorption of silver cations onto Lactococcus lactis and Lactobacillus casei isolated from dairy products
Source: PLoS One. 2017 Mar 31;12(3):e0174521. doi: 10.1371/journal.pone.0174521 (PMC5375156; doi:10.1371/journal.pone.0174521)

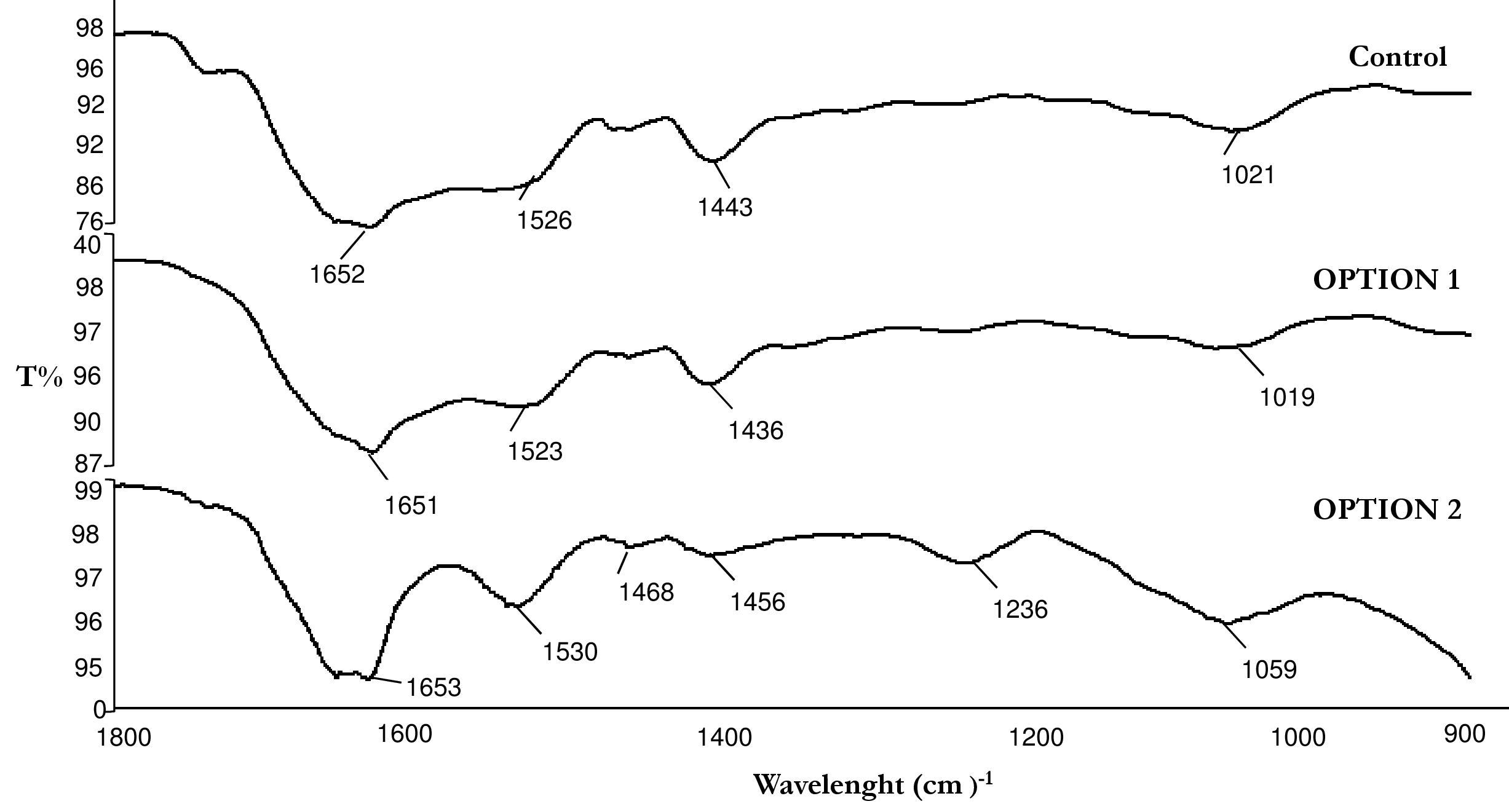

Supplement: S1 Fig — (TIF) [file pone.0174521.s002.tif]

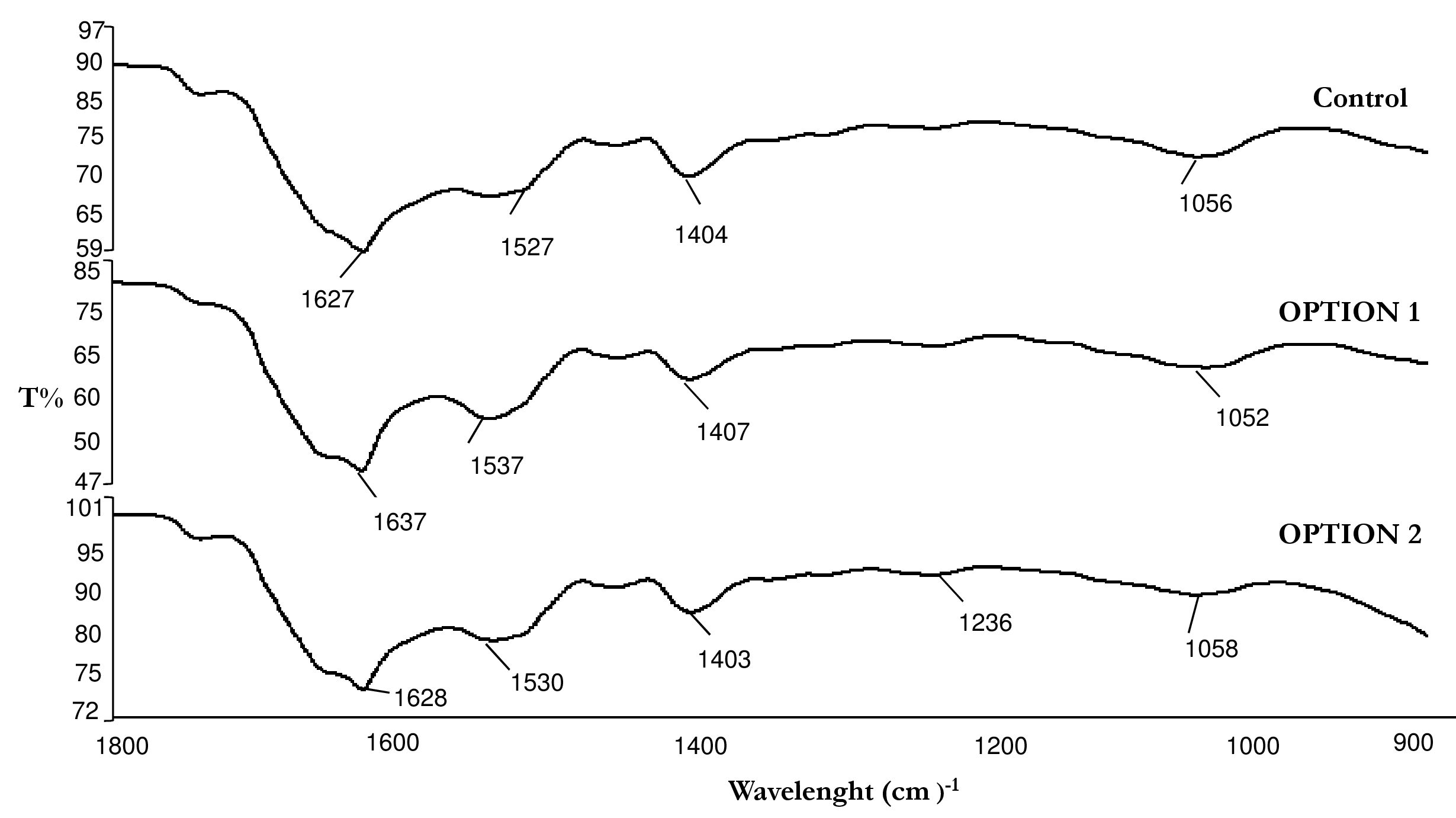

Supplement: S2 Fig — (TIF) [file pone.0174521.s003.tif]

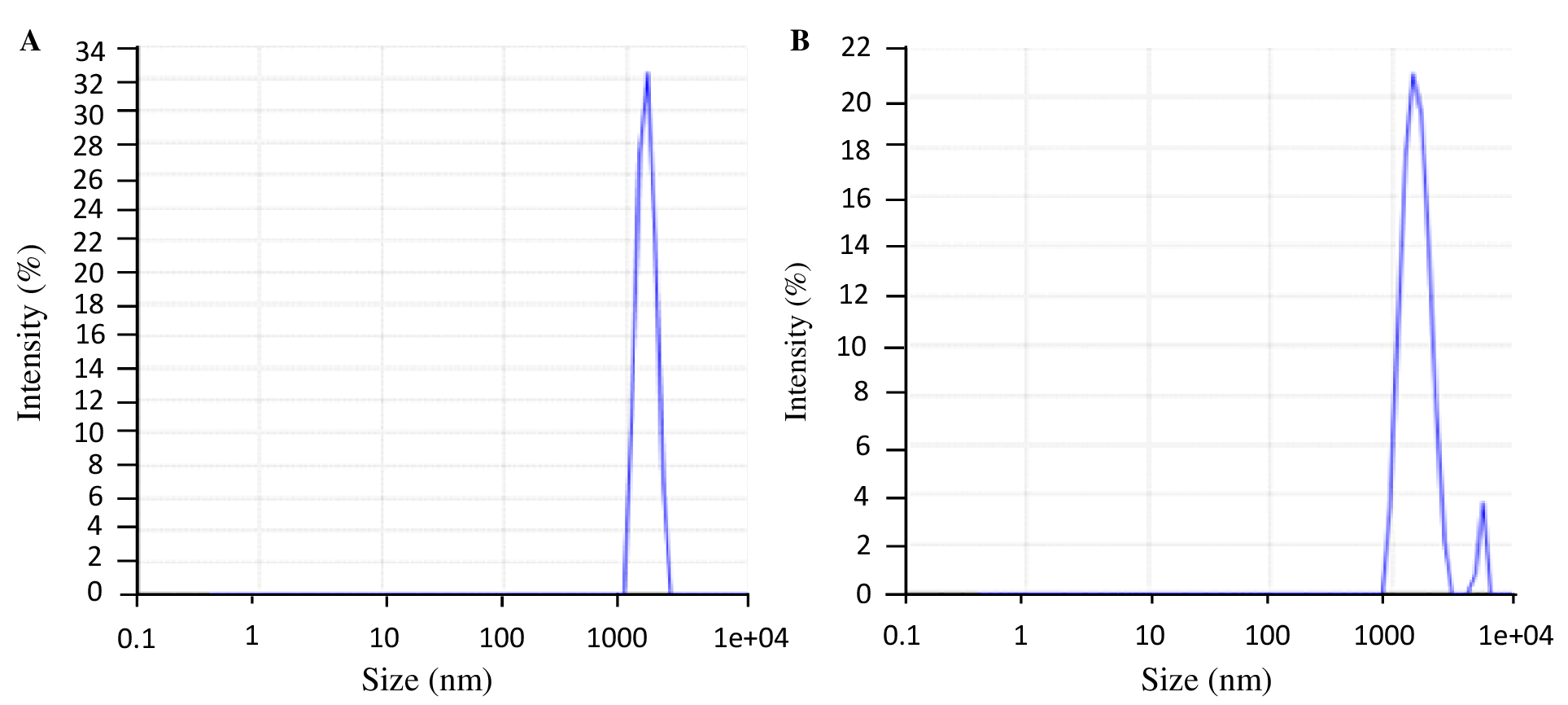

Supplement: S3 Fig — (TIF) [file pone.0174521.s004.tif]
